# The clinicopathological significance of neurogenesis in breast cancer

**DOI:** 10.1186/1471-2407-14-484

**Published:** 2014-07-04

**Authors:** Qianqian Zhao, Yan Yang, Xizi Liang, Guangye Du, Liwei Liu, Lingjuan Lu, Junbo Dong, Hongxiu Han, Guohua Zhang

**Affiliations:** 1 Department of Pathology, Shanghai Third People’s Hospital, Shanghai Jiaotong University School of Medicine, 280 Mohe Road, Shanghai 201999, China; 2 Department of Pathology, Shanghai First Maternity and Infant Healthy Hospital, Tongji University, 536 Changle Road, Shanghai 200040, China; 3 Department of Physiology, Shanghai Jiaotong University School of Medicine, 280 South Chongqing Road, Shanghai 200025, China

**Keywords:** Neurogenesis, Breast cancer, Nerve density, Angiogenesis

## Abstract

**Background:**

Recent reports support a novel biological phenomenon about cancer related neurogenesis. However, little is known about the clinicopathological significance of neurogenesis in breast cancer.

**Methods:**

A total of 196 cases, including 20 of normal tissue, 14 of fibroadenoma, 18 of ductal carcinoma in situ (DCIS) and 144 of invasive ductal carcinoma (IDC) of the breast were used. The tissue slides were immunostained for protein gene product (PGP) 9.5 and S 100 to identify nerves. The correlation between the expression of PGP 9.5 and clinicopathological characteristics in IDC of the breast was assessed.

**Results:**

While the PGP 9.5 positive nerve fibers are identified in all cases of normal breast tissue controls and in the tumor stroma of 61% (89/144) cases of invasive ductal carcinomas, PGP 9.5 positive nerve fibers are not seen in the tumor stroma of cases of fibroadenoma and DCIS. The percentage of tumors that exhibited neurogenesis increased from tumor grade I to tumor grade II and III (29.4% vs 71.8%, p < 0.0001). In addition, patients with less than 3 years of disease-free survival tended to have a higher positive expression of PGP 9.5 compared to patients with an equal or more than 3 years of disease-free survival (64.8% vs 46.7%, p = 0.035). Furthermore, moderate/strong expression of PGP 9.5 was found to be significantly related to microvessel density (MVD, p = 0.014). Interestingly, PGP 9.5 expression was significantly associated with higher MVD in the ER-negative (p = 0.045) and node-negative (p = 0.039) subgroups of IDC of the breast.

**Conclusions:**

This data indicates that neurogenesis is associated with some aggressive features of IDC including tumor grade and patient survival as well as angiogenesis, especially in ER-negative and node-negative subtypes of IDC of the breast. Thus, neurogenesis appears to be associated with breast cancer progression and may play a role in therapeutic guidance for patients with ER-negative and node-negative invasive breast cancer.

## Background

Tumor-stromal interactions are critical to cancer development. For example, angiogenesis, inflammation, matrix remodeling and perineural invasion (PNI). It is well known that PNI is a poor prognostic factor in malignancies like prostate [1], head and neck [2], and pancreatic cancer [3]. However, recent reports have described a novel biological phenomenon that active neurogenesis occurs in cancer, which indicates specific interactions between cancer cells and the existence of nerve fibers other than PNI [4-6].

Entschladen F et al proposed the hypothesis that tumors may initiate their own innervation by the release of neurotrophic factors similar to that of angiogenesis [7]. It is likely that tumor neurogenesis is related to metastasis, since the ingrown nerve endings can release neurotransmitters which enhance the metastasis development. Ayala GE et al first described cancer-related neurogenesis and its putative regulatory mechanism in prostate cancer. This study provides strong evidence that neurogenesis does occur in prostate cancer and increased nerve density has been found in tumors compared with normal peripheral zone [4]. In addition, Albo D et al reported that neurogenesis in colorectal cancer appeared to play a critical role in colorectal cancer progression [5]. Accumulating evidence indicates that neuronal system-dependent facilitation of tumor angiogenesis and tumor growth by calcitonin gene-related peptide [8] or nerve growth factor [9] occurred in breast cancer. However, the role of neurogenesis in breast cancer is unclear.

We hypothesize that neurogenesis is important in breast cancer progression. Therefore, we investigated whether neurogenesis occurs in breast cancer, if so; we evaluated its clinicopathological significance.

## Methods

### Clinical specimens

One hundred and ninety-six cases were retrieved from the files of the Departments of Pathology in Shanghai Third People's Hospital, Shanghai Jiaotong University School of Medicine and Shanghai First Maternity and Infant Healthy Hospital, Tongji University. The most histological type was invasive ductal carcinoma (144 cases), followed by ductal carcinoma in situ (DCIS, 18 cases), fibroadenoma (14 cases) and then normal breast tissue (20 cases). The pathological parameters, including tumor size, differentiation and the presence of nodal metastasis, were carefully reviewed. The histological grade and stage were evaluated by a modified Bloom-Richardson grading system and American Joint Committee on Cancer (AJCC), respectively. Out of 144 invasive ductal carcinoma (age range = 32-71 years; average age = 48.06 years), 62.5% (90/144) were equal to/more than 3 years of disease free survival. All the patients with IDC received adjuvant therapy. In addition, the patients with distant metastasis were not enrolled in this study. One representative paraffin block from each case was used for the study. The study was approved by the Ethical Review Boards of Shanghai Third People's Hospital, Shanghai Jiaotong University School of Medicine and Shanghai First Maternity and Infant Healthy Hospital, Tongji University. No consent from patients involved in this study was needed because the required consent was waived by Ethical Review Boards. The information about patients involved in this study is kept confidential at all times.

### Immunohistochemistry

Immunohistochemical assays were performed on formalin-fixed paraffin-embedded tissues. Sections (5 μm thick) were cut, deparaffinized in xylene and rehydrated in graded alcohols. Slides were boiled in citrate buffer (pH 6.0) at 95 ~ 100°C for 5 minutes and were cooled for 20 minutes. Endogenous peroxide was blocked by 3% hydrogen peroxide in methanol for 10 minutes. Sections were incubated with rabbit anti-human PGP 9.5 (1:500, DAKO, Carpinteria, CA, USA), rabbit anti-human S100 (1:200, DAKO, Carpinteria, CA, USA) and mouse anti-human CD34 antibodies overnight at 4°C. Immunohistochemical staining was performed using EnVision + HRP DAB system (DAKOCytomation, Carpinteria, CA, USA). All sections were counterstained with Meyer’s Hematoxylin. The sections processed without the primary antibodies were used as negative control.

### Interpretation of immunohistochemical staining

A pathologist read all immunostained slides. Each slide was marked at points in which positive PGP 9.5/S100 immunostaining was shown. A single digital image was created with the Olympus BLISS HD virtual microscopy system at × 400 magnification. The diameters of nerve fascicles were measured with the Optimas 6 Image Analysis Suite (Optimas Corp.) Most of the diameters of nerve fascicles were less than 100 μm (94.7%). Nerve density was evaluated by counting the number of nerve fascicles with diameters of < 100 μm in 20 continuous fields at × 200 magnification. Nerve density results were grouped into 3 categories: 1) negative, no nerve fascicles or nerve fibers, 2) weak expression, 1 to 10 nerve fascicles, and 3) moderate/strong expression, > 10 nerves fascicles. Intratumoral microvessel density (MVD) was recorded by counting CD34-positive vessels in the most vascularized area in four × 200 fields [10]. Blood vessels with a lumen diameter exceeding approximately eight red blood cells were excluded. For estrogen receptor (ER) and progesterone receptor (PR), we defined cases with more than 5% positive tumor cells of moderate intensity as positive. Immunohistochemistry results were analyzed by three independent pathologists under a multihead microscope in cases of disagreement.

### Statistical analysis

All statistical analyses were carried out using SPSS software (SPSS Ver. 11.0, USA). Some data was presented as absolute numbers and percentages, other data was presented as mean ± SD. *χ*^2^-test was used to examine the association between PGP 9.5 expression and the various clinicopathological characteristics. The relationship between PGP 9.5 expression and MVD was evaluated using the Mann-Whitney test. Reported *P*-values less than 0.05 were considered as significant.

## Results

### Neurogenesis in breast cancer

**Figure 1:**
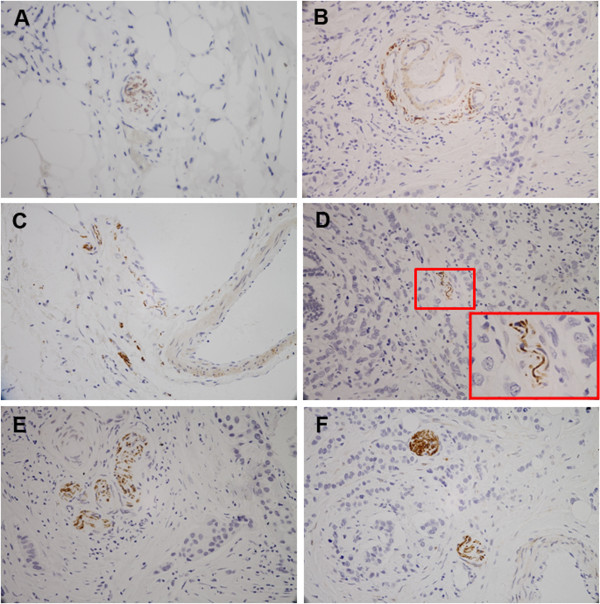
**Photomicrograph representatives of nerves in normal breast tissue (A, PGP 9.5 expression) and in invasive ductal carcinoma of the breast (B-E, PGP 9.5 expression; F, S 100 expression).** The scattered fine nerve fibers were seen in the stroma **(B-C)** and most of them surrounded the blood vessels **(B)**. Some fine nerve fibers were sporadically located among the cancer cells **(D)**, the high-magnification view of the area in the box in photomicrograph D is the fine nerve fibers. In most of the cases the fragmented nerve fascicles were distributed in the stroma of tumor **(E-F)**.

PGP 9.5 or S100 expression was identified in normal breast tissue control cases and in high percentage of breast cancer cases. The pattern of neurogenesis in IDC was shown in Figure 1. In the most of cases the fragmented nerve fascicles were distributed in the tumor stroma. In some cases, the scattered fine nerve fibers were seen surrounding the blood vessels. Furthermore, some fine nerve fibers were sporadically located around the cancer cells. Nerve fibres were found in all the normal breast tissue control cases. However, there is no PGP 9.5 positive nerve fiber identified in the stroma of cases of fibroadenoma and DCIS. Overall, 61.8% (89/144) of invasive ductal carcinoma cases exhibited evidence of neurogenesis. PGP 9.5 positive nerve fibers were observed in all normal breast tissue controls (Table 1). There is no difference in diameters of the nerve fibers between normal breast tissue control (23.4 ± 8.2 μm) and in invasive ductal carcinomas (20.8 ± 10.4 μm).

**Table 1:** PGP 9.5 expression in different histological types of the breast

		**PGP 9.5 expression**			
**Diagnosis**	**Case no.**	**Negative**	**Weak**	**Moderate/strong**	**Positive rate**
IDC	144	55	54	35	61.8%
DCIS	18	18	0	0	0
Fibroadenoma	14	14	0	0	0
Normal	20	0	18	2	100%

*IDC* Invasive ductal carcinoma, *DCIS* Ductal carcinoma in situ.

### The relationship between neurogenesis and clinicopathological characteristics in IDC of the breast

While analyzing invasive ductal carcinomas of different MBR grade, we found that PGP 9.5 positive rate was 29.4% (10/34) in grade I cases, but PGP 9.5 positive rate was 71.8% (79/110) in grade II and III cases, the difference was statistically significant (p < 0.0001). The PGP 9.5 positive rate was significantly higher in cases of less than 3 years of disease-free survival (64.8%, 35/54) compared to cases of equal or more than 3 years of disease-free survival (46.7%, 42/90; p = 0.035). However, no significant difference in PGP 9.5 expression was found between tumor groups of different stage (stage I: 56.5%; stage II: 63.5%; stage III: 61.8%). Likewise, no significant difference of PGP 9.5 expression was found in cases of different lymph node, ER and PR status (Table 2).

**Table 2:** Relationship between clinicopathological data and PGP 9.5 expression in invasive ductal carcinoma of the breast

		**PGP 9.5 expression**		
	**No. case**	**Negative**	**Positive**	**P-value**
**Tumor size**				**P = 0.319**
≤ 2 cm	86	30 (34.9%)	56 (65.1%)	
> 2 cm	58	25 (43.1%)	33 (56.9%)	
**Tumor grade**				**P < 0.0001**
I	34	24 (70.6%)	10 (29.4%)	
II - III	110	31 (28.2%)	79 (71.8%)	
**Tumor stage**				**P = 0.834**
I	23	10 (43.5%)	13 (56.5%)	
II	74	27 (36.5%)	47 (63.5%)	
III	47	18 (38.2%)	29 (61.8%)	
**LN metastasis**				**P = 0.825**
Negative	75	28 (37.3%)	47 (62.7%)	
Positive	69	27 (39.1%)	42 (60.9%)	
**ER**				**P = 0.399**
Negative	80	33 (41.3%)	47 (58.8%)	
Positive	64	22 (34.4%)	42 (65.6%)	
**PR**				**P = 0.801**
Negative	70	26 (37.1%)	44 (62.9%)	
Positive	74	29 (39.2%)	45 (60.8%)	
**Disease free survival**				**P = 0.035**
< 3 years	54	19 (22.2%)	35 (64.8%)	
≥ 3 years	90	48 (53.3%)	42 (46.7%)	

*LN* Lymph node, *ER* Estrogen receptor, *PR* Progesterone receptor.

### The relationship between neurogenesis and angiogenesis in IDC of the breast

As shown in Table 3, moderate/strong PGP 9.5 expression (more than 10 nerves) was found to be significantly related with tumor MVD (p = 0.014). Intriguingly, analysis of subgroups of ER and node status revealed that moderate/strong expression of PGP 9.5 was significantly associated with higher MVD in the ER-negative (p = 0.045) and node-negative (p = 0.039) subgroups. No significant association was found between PGP 9.5 expression and MVD in ER-positive and node-positive subgroups.

**Table 3:** Relationship between the PGP 9.5 expression and microvessel density (MVD) according to the estrogen receptor status and lymph node involvement in invasive ductal carcinoma of the breast

	**No. cases**	**MVD**	**P value**
**PGP 9.5 expression total**			**P = 0.014**
Weak	54	37.15 ± 10.30	
Moderate/strong	35	43.11 ± 11.30	
**ER-positive**			**P = 0.148**
Weak	27	38.22 ± 10.64	
Moderate/strong	15	43.40 ± 11.33	
**ER-negative**			**P = 0.045**
Weak	27	36.07 ± 10.03	
Moderate/strong	20	42.90 ± 11.56	
**LN-positive**			**P = 0.162**
Weak	24	39.13 ± 8.53	
Moderage/strong	18	43.56 ± 11.63	
**LN-negative**			**P = 0.039**
Weak	30	35.37 ± 11.02	
Moderate/strong	17	42.64 ± 11.27	

## Discussion

Our data show that some degree of neurogenesis occurs in the invasive ductal carcinoma when compared to fibroadenomas and DCIS. This indicates that cancer-related neurogenesis does occur in breast cancer, which is similar to previously reported observation that active neurogenesis occur in prostate cancer [4], colorectal cancer [5], esophageal and cardiac carcinoma [11], tumors of the human urinary bladder [12] and choroidal melanoma [13]. Taken together, this novel phenomenon that cancer initiates its own innervations may be universal.

Neuroepithelial interactions occur at several stages of oncogenesis. PNI is the most obvious and well studied [14,15]. Cancer-related neurogenesis most likely facilitates PNI, which would then become the second step of neuroepithelial interactions in tumor. Understanding of the cancer-related neurogenesis may be of help in developing cancer-related therapies in breast cancer.

Ayala and colleagues [4] recently reported that nerve density in prostate tissues was higher in cancer and premalignant specimens compared to normal prostate tissues. Also, neurogenesis was correlated with features of aggressive prostate cancer and with recurrence in prostate cancer. In addition, Albo and colleagues [5] demonstrated neurogenesis in colorectal cancer as a marker of aggressive tumor behavior and poor outcomes. Our data show that some degree of neurogenesis occurs in IDC, but not in DCIS and fibroadenoma of the breast. Of IDC of the breast, neurogenesis is correlated with tumor grade and disease-free survival. This indicates that neurogenesis is functionally significant in human disease and involved in the progression of breast cancer.

The initial observation that a tumor stimulates and nurtures the development of blood vessels for its own nourishment was made over 30 years ago [16]. Since then, a plethora of studies have unraveled the mechanisms of this phenomenon called neoangiogenesis, and several promising anti-angiogenic drugs have been developed [17]. In the last 10 years, several studies have thoroughly demonstrated that vascules and neurons share molecular tools and strategies during their networking [18-20]. Our data suggests a significant relationship between neurogenesis and angiogenesis in breast cancer. Interestingly, our study shows that higher nerve density is significantly associated with MVD in ER-negative and node-negative groups of IDC. These findings may open a future direction for the targeted therapy for these groups of ER negative and node-negative IDC. Further studies will explore the mechanisms underlying the interaction between neurogenesis and angiogenesis in breast cancer.

## Conclusions

This data indicates that neurogenesis is associated with some aggressive features of IDC including tumor MBR grade and patient survival. It is also associated with tumor angiogenesis, particularly in the ER-negative and node-negative subtypes of IDC. Thus, neurogenesis appears to be associated with breast cancer progression and may play a role in therapeutic guidance for patients with ER-negative and node-negative invasive breast cancer.

## Abbreviations

DCIS: Ductal carcinoma in situ; IDC: Invasive ductal carcinoma; PGP: 9.5 Product growth protein; MVD: Microvessel density; ER: Estrogen receptor; PR: Progesterone receptor; AJCC: American Joint Committee on Cancer; PNI: Perineural invasion.

## Competing interests

The authors declare that they have no competing interests.

## Authors’ contributions

QZ and YY performed the study, interpreted data and drafted the manuscript. XL, GD and LL^1^ helped to conduct the statistical analyses and were involved in drafting the manuscript. LL^2^ and JD helped to carry out the immunoassays. HH and GZ conceived of the study, and participated in its design and coordination and helped to draft the manuscript. In cases of discrepant assessments, HH, YY and GD discussed to come to an agreement. All authors read and approved the final manuscript.

## Pre-publication history

The pre-publication history for this paper can be accessed here:

http://www.biomedcentral.com/1471-2407/14/484/prepub
